# Multiple, Unilateral Lisch Nodules in the Absence of Other Manifestations of Neurofibromatosis Type 1

**DOI:** 10.1155/2011/854784

**Published:** 2012-01-23

**Authors:** E. G. Adams, K. M. A. Stewart, O. A. Borges, T. Darling

**Affiliations:** ^1^Department of Dermatology, Walter Reed National Military Medical Center, Bethesda, MD 20889, USA; ^2^Department of Dermatology, Naval Health Clinic New England, Newport, RI 02841, USA; ^3^Department of Ophthalmology, Naval Health Clinic New England, Newport, RI 02841, USA; ^4^Department of Dermatology, Uniformed Services University of the Health Sciences, Bethesda, MD 20814, USA

## Abstract

Lisch nodules associated with Neurofibromatosis Type 1 (NF1) are usually multiple and bilateral in nature. Here, we report a 21-year-old healthy, Caucasian female who was diagnosed with multiple, unilateral Lisch nodules during routine eye examination. A thorough history and physical examination revealed no other signs of NF1. We diagnosed the rare occurrence of numerous, unilateral Lisch nodules in the absence of additional features of NF1 in our patient and provide a discussion concerning the differential diagnosis of Lisch nodules as well as the potential genetic explanation of this finding.

## 1. Introduction

Lisch nodules are the most common ophthalmologic manifestation of NF1 and are included in the clinical diagnostic criteria for NF1. They are not diagnostic when present as an isolated finding. Multiple Lisch nodules associated with NF1 are almost always bilateral. Multiple, unilateral Lisch nodules occur rarely, and somatic mosaicism may explain their presence. Prenatal genetic counseling should be offered to patients with numerous, unilateral Lisch nodules.

## 2. Case Presentation

We present a case of a 21-year-old healthy, Caucasian female diagnosed with multiple, unilateral Lisch nodules during routine eye examination by an ophthalmologist who then referred the Patient to Dermatology to evaluate for cutaneous signs of neurofibromatosis type 1 (NF1). Her medical history revealed a full-term, uncomplicated birth and an episode of patellar dislocation. She denied history of skin growths, scoliosis, birthmarks, learning disabilities, seizures, and growth or developmental delays. There was no family history of mental retardation, cancer, pigmentary abnormalities, or genetic diseases, including NF1.

**Figure 1:**
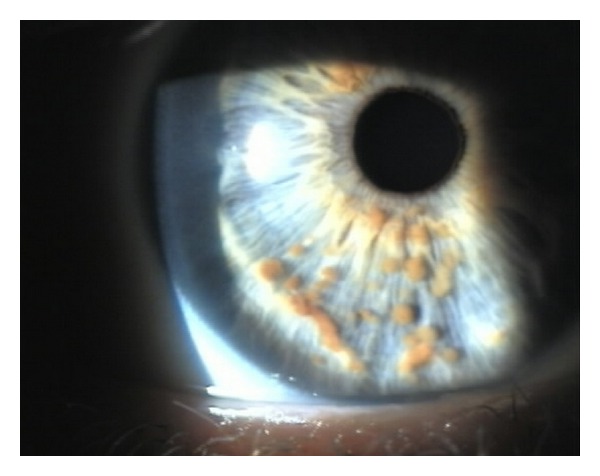
Multiple small, oval, yellow-brown papules (Lisch nodules) in the right iris.

On physical examination, blood pressure was normal. A solitary 5 mm hypopigmented macule was observed on her back under Wood's light illumination. There were no neurofibromas, café-au-lait macules (CALMs), axillary, or inguinal freckling. Head circumference was within normal range for an adult female (56 cm), and neither hypertelorism nor ear abnormalities were present. Slit-lamp examination revealed multiple small, oval, yellow-brown, fleshy papules randomly spaced on the inferior surface of her right iris consistent with Lisch nodules (Figure 1). There was no associated underlying nevus; vision, fundoscopic examination and intraocular pressures were unremarkable.

## 3. Discussion

Lisch nodules are the most common ophthalmologic manifestation of NF1 and are included in the clinical diagnostic criteria for NF1 [1]. Histologically, they are melanocytic hamartomas, presumably of neural crest origin, similar to other cutaneous characteristics of NF1 [2]. They are not diagnostic when present as an isolated finding, but iris nodules occur predominantly in individuals with NF1 (90–100% of adults with NF1) [2]. The differential diagnosis of Lisch nodules includes iris mammillations, multiple iris nevi, iris melanoma, Cogan-Reese (ICE) syndrome, granulomatous iritis, iris cysts, retinoblastoma, Brushfield flecks, and other malformations [3, 4]. In our otherwise healthy patient, iris mammillations and multiple iris nevi were mainly considered. Iris mammillations are frequently confused with Lisch nodules and are characterized by regularly spaced, deep brown, and smooth conical iris elevations [5]. Often found in more deeply pigmented ethnic groups, they are seen in association with oculodermal melanosis and may be an external manifestation of ocular hypertension or intraocular malignancy [5]. Iris nevi present as flat, or minimally elevated, densely pigmented lesions with blurred margins [6]. These can be differentiated from Lisch nodules by slit-lamp examination as Lisch nodules are well-defined, dome-shaped elevations rising from the surface of the iris [6].

Unilateral Lisch nodules are rare. They have been reported in cases of segmental neurofibromatosis, found associated with other pigmentary changes or neurofibromas. To our knowledge, only five other cases of Lisch nodules without other clinical evidence of NF1 have been reported [2, 4, 7]. Only one other reported case of *numerous*, *unilateral* Lisch nodules in the absence of additional features of NF1 exists [7].

Possible genetic explanation of isolated, unilateral Lisch nodules is also of interest. NF1 is typically inherited in an autosomal dominant fashion, but approximately 50% of cases represent a new, sporadic mutation. Somatic mosaicism accounts for many sporadic NF1 cases; however, the clinical phenotype reflects the timing of the somatic mutation as well as the involved tissues [8]. Ruggieri and Huson subdivided the clinical presentation of mosaicism into generalized disease, localized or segmental disease, and pure gonadal mosaicism [8]. Segmental disease is caused by late-stage mutations in the NF1 gene during embryogenesis and, in a very limited manner, could explain the development of unilateral Lisch nodules without other clinical characteristics of NF1. Genetic testing of the affected tissue might detect such mutation; however, an iris biopsy for a molecular study of the NF1 gene would likely cause excessive morbidity in an otherwise healthy patient. Brain magnetic resonance imaging might detect further segmental involvement of neurologic tissue.

Our patient did not meet criteria for NF1 and declined genetic testing as well as further imaging studies. Although isolated Lisch nodules are rare, their presence warrants a thorough evaluation for NF1 [4]. Additionally, patients with segmental disease have been reported to bear children with NF1, indicating gonadal involvement [8]. These patients are termed gonosomal mosaics [8], and prenatal counseling should be considered.

##  Disclousure

The views expressed in this paper are those of the author and do not necessarily reflect the official policy or position of the Department of the Navy, Army, Department of Defense, nor the U.S. government. We certify that all individuals who qualify as authors have been listed; each has participated in the conception and design of this work, the analysis of data (when applicable), the writing of the document, and the approval of the submission of this version, that the paper represents valid work, that if we used information derived from another source, we obtained all necessary approvals to use it and made appropriate acknowledgments in the paper, and that each takes public responsibility for it.

##  Conflict of Interests

The authors have no conflict of interests to declare.
